# Supplementary material for: Arabidopsis V-ATPase d2 Subunit Plays a Role in Plant Responses to Oxidative Stress
Source: Genes (Basel). 2020 Jun 25;11(6):701. doi: 10.3390/genes11060701 (PMC7349310; doi:10.3390/genes11060701)
Supplement: Supplementary file 1 [file genes-11-00701-s001.zip › Supplementary Figure S1.docx]

**LP+RP LB+RP**

**Col-0 #1 #2 #3 #4 M Col-0 #1 #2 #3 #4 --**


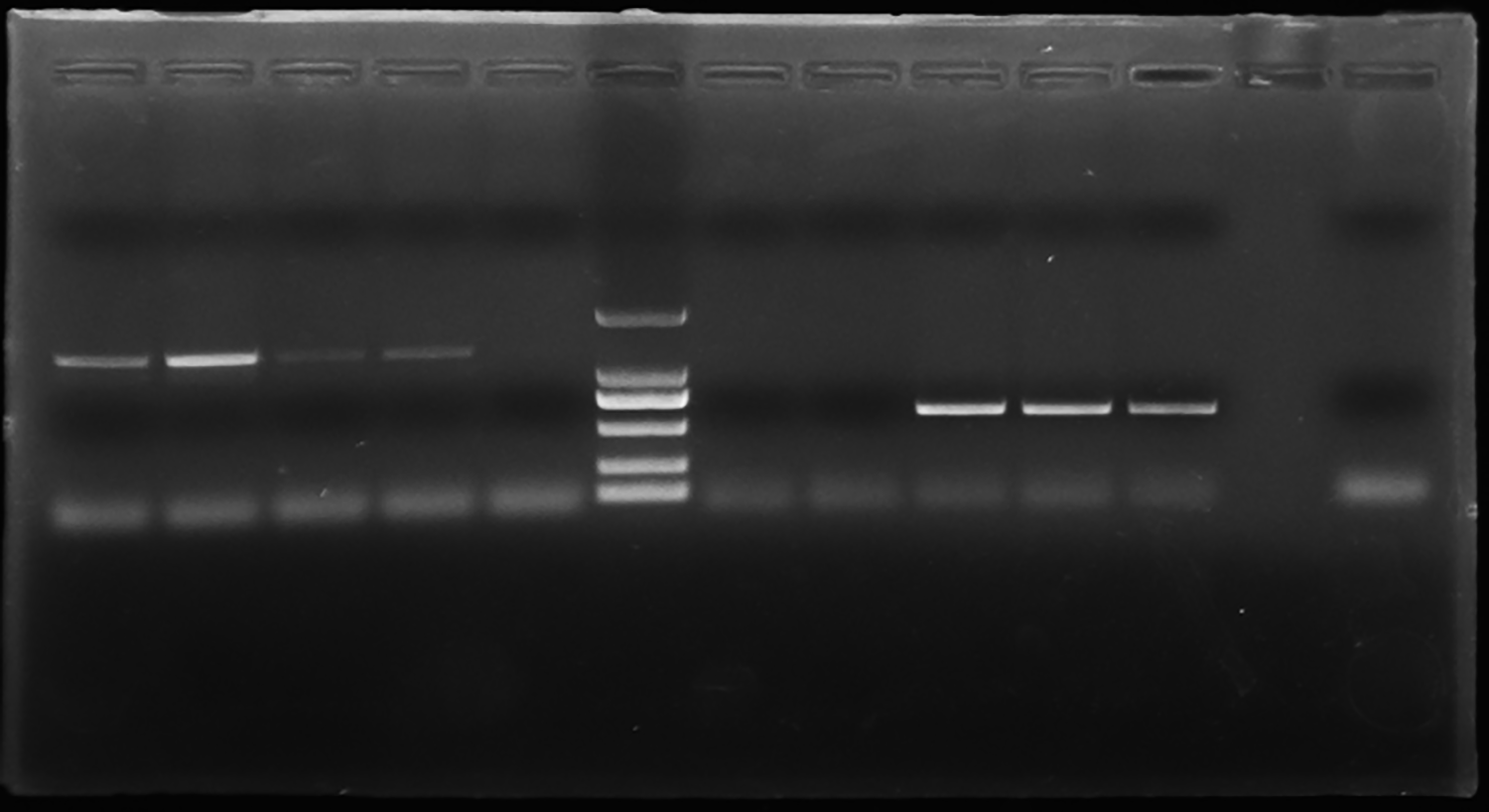


**Supplementary** **Figure S1.** PCR [detection](http://xueshu.baidu.com/usercenter/paper/show?paperid=aa2df47bdbf11de562d9d54e8d15e54f&site=xueshu_se) of T-DNA insertion mutants of *AtVHA-d2* gene.

M: DL2000 DNA marker; #1-#4: putative mutant lines; Line #4 indicated by the red box is a homozygous mutant line; **--**: negative control
